# Polypharmacy as a Chronic Condition: A Diagnostic Mindset for Safer and Smarter Care

**DOI:** 10.3390/jcm14207388

**Published:** 2025-10-19

**Authors:** Waseem Jerjes, Azeem Majeed

**Affiliations:** Department of Primary Care and Public Health, Faculty of Medicine, Imperial College London, 90 Wood Ln, London W12 0BZ, UK; a.majeed@imperial.ac.uk

**Keywords:** polypharmacy, chronic condition management, medication review, prescribing cascades, anticholinergic burden, deprescribing, primary care

## Abstract

Polypharmacy is typically seen as an unavoidable consequence of multimorbidity and aging, with clinicians addressing complex medication lists unsystematically. In this perspective, we argue that polypharmacy should be managed as a chronic condition. Like diabetes or hypertension, for example, the medication burden shows persistence, progression in its absence despite active management, predictable complications (such as falls, delirium, renal injury, functional decline), and a need for structured surveillance. We introduce a pragmatic diagnostic framework that moves beyond pill counts to modality-agnostic, regimen-level risk across prescribed and non-prescribed medicines. Diagnosis rests on prolonged exposure, composite burden indices (e.g., anticholinergic/sedative load), medication-related complications or prescribing cascades, and the need for a planned review. As biologics, gene therapies and long-acting formulations can lower tablet numbers while increasing monitoring, administration, and interaction complexity. We treat polypharmacy as cumulative pharmacodynamic and operational burden. We advocate stage matched care with unique, functional aims—decreasing the harmful burden instead of mass deprescribing—and position a structured medication review as the standard for polypharmacy with support from pharmacists, shared decision making, and safety netted taper plans. The framework fosters patient-centred care, embedding continuity and equity, and outlines a concise outcome set that integrates pharmacometric measures with patient-reported function and treatment burden. At the systems level, the framework enables registries, recall systems, decision support, and audit/feedback mechanisms to shift from sporadic medication list clean-up to a structured, measurable long-term program. Redefining polypharmacy in this way aligns clinical practice, education, and policy with real-world evidence, fostering a cohesive pathway to safer, streamlined, and more patient-centred care in community settings.

## 1. Introduction

Polypharmacy—generally defined as concomitant prescribing of five or more medications—is increasingly a routine part of daily clinical practice, occurring more often among older people and those with multimorbidity and frailty [1,2]. Its development is a manifestation of advances in disease-specific therapy but carries with it unavoidable side effects that single disease guidelines were never designed to manage [1,2,3].

Among frail older adults, pooled prevalence estimates place polypharmacy at 59% and hyperpolypharmacy at 22%; rates are highest in hospital settings (71%) and in European studies (68%) [1]. In primary care, a scoping review of 157 articles found no consensus definition of “problematic” polypharmacy and substantial heterogeneity in identification and management approaches [2]. A systematic review of reviews synthesising 26 reviews (230 unique studies) showed the most consistent associations with hospitalisation and inappropriate prescribing, with mixed findings for other outcomes [3]. In a 6-year cohort of community-dwelling adults ≥ 70 years (*n* = 592), 26.9% experienced at least one adverse drug reaction; risk rose with medication count (adjusted OR 1.81 for 5–9 drug classes; 3.33 for ≥10), and 3.8% of all ADRs (Adverse Drug Reactions) led to emergency admission [4].

The impacts of polypharmacy include increased ADRs, drug–drug interactions, functional decline, falls, and diminished quality of life [1,3]. Nonetheless, service models and clinical reasoning are typically disease oriented, increasing treatment disease by disease and guideline by guideline. Such a compartmentalised approach encourages cascade prescribing, where new medication is started to treat side effects of medication already being taken, leading to further complications [5,6]. The prevailing focus on “appropriate versus inappropriate prescribing” is overly simplistic, emphasising individual prescription decisions while overlooking the long-term dynamics of a drug regimen, such as its persistence, potential for uncontrolled escalation, and risk of complications requiring further monitoring [5,6].

In this perspective, we argue that polypharmacy should be recognised and managed as a chronic condition. Like diabetes or hypertension, it has discernible diagnostic features (persistence and progression), predictable complications (ADRs, functional loss, falls), and a need for ongoing monitoring with active management. Our assertion is aligned with recent science on deprescribing—describing processes, results, and patient-centred decision making, with syntheses of evidence advancing longitudinal, team-centred systems of care in primary care [7,8]. It also aligns with conceptual and practice-based expositions suggesting the limits of reactive, episodic deprescribing and a need for proactive, condition-like care pathways centred on medication burden as part of a differential diagnosis [9,10].

Defining polypharmacy thus moves the emphasis from an audit of all medicines (prescribed and non-prescribed) to longitudinal stewardship: continuity of care, collaborative decision making, planned medication review, collaboration with pharmacists, and scheduled regime “maintenance”, instead of reduction of crises [8,11]. It also extends the scope beyond pharmacology to social determinants which modify experience and medication burden outcomes—such as health literacy, isolation, and access barriers—with a focus on person-centred care; operationally this includes registries with social-risk flags, translated plain-language materials, interpreter use, carer involvement, and outreach reviews for housebound or socially excluded patients [12].

We used the current practice of primary care, frailty medicine, and psychiatry evidence to delineate (i) why polypharmacy is a chronic condition by definition, (ii) a diagnostic strategy to identify medication burden as a first aetiology, and (iii) practice, education, digital solutions, and policy applications. Our aim is a unifying framework to transition clinicians, educators, and systems leaders from temporary solutions to sustained, condition-level management of one of our most pressing safety concerns. A graphical abstract summarises the framework.

## 2. Concept and Working Definition

To frame polypharmacy as a chronic condition is more than just a change in definition; it enshrines a predictable risk pattern over time that is quantifiable with evidenced tools, with pre-specified action thresholds linked to stage and review cadence, and responsive to planned long-term management. An analogous condition-level framework also emphasises diagnosis, staging, and “treat to target” strategies missing in single disease guidelines. In the next section, we make the practical case for that framework: how practitioners can frame the problem, stage its intensity, and manage it with comparable intent to other long-term conditions, without identifying the goal with pill reduction. This approach calls upon established instruments of measurement (e.g., Drug Burden Index, scales of Anticholinergic Cognitive Burden, medication review tools) and support for deprescribing processes and facilitating systems [13].

## 3. Diagnostic Domains and Staging

From aggregation to diagnosis, polypharmacy is often reduced to a mere count of medications, but its clinical burden hinges on the specific drugs combined, their interactions, and the individual patient’s characteristics. A condition level definition would move a step beyond thresholds (≥5 medications) to a diagnosis construct that captures: (i) persistence (medication combinations that persist over months), (ii) progression (escalation following an acute episode or layering of guidelines), (iii) predictable complications (falls, cognitive shift, orthostatic symptoms, gastrointestinal distress), and (iv) an indication for structured review. Two things then follow. First, clinicians should regularly assess the pharmacological burden and interaction risks, moving beyond simply counting medications. Second, diagnosing polypharmacy should trigger a structured plan for ongoing review and follow-up, rather than a passive “polypharmacy noted” entry in the problem list. Tools such as sedative and anticholinergic burden indices and regimen complexity scores provide practical, evidence-based starting points for this process and can be integrated into routine clinical reviews [13].

**Figure 1:**
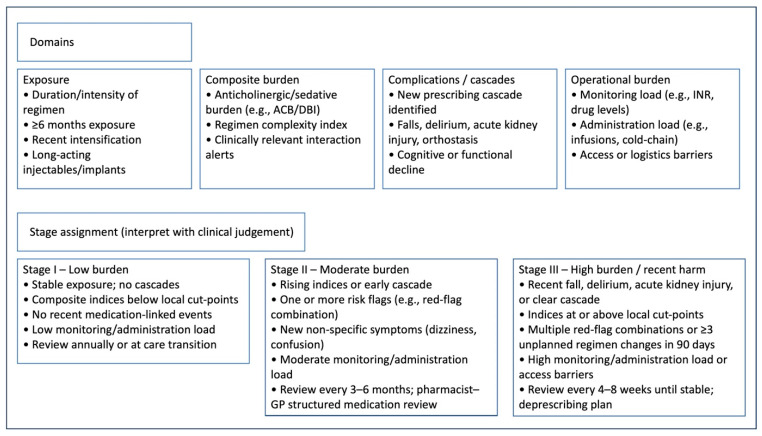
Diagnostic domains and stage assignment for “polypharmacy as a chronic condition”. Schematic showing four diagnostic domains—exposure, composite burden (e.g., anticholinergic/sedative load, regimen complexity), complications/prescribing cascades, and operational burden (monitoring/administration/logistics)—and how findings inform stage assignment (low, moderate, high/recent harm). Framework is modality-agnostic and includes prescribed and non-prescribed agents (OTC, herbal/supplements, biologics, long-acting/implantable therapies). Measures shown are examples; apply locally validated indices and thresholds (e.g., ACB/DBI cut-off points) and interpret with clinical judgement.

Action should be triggered by any of the following: a new fall, delirium/confusion, orthostatic symptoms, acute kidney injury, cognitive or functional decline, a suspected prescribing cascade, red-flag combinations (for example benzodiazepines plus opioids), anticholinergic or sedative burden above the locally agreed cut-off point, or ≥3 unplanned regimen changes within 90 days. Routine reconciliation should explicitly include OTC, herbal and supplement use (for example brown-bag reviews and community pharmacy medication summaries) with prompts to reassess potential interactions and cumulative sedative/anticholinergic load. Where validated cut-off points exist (for example, Anticholinergic Cognitive Burden or Drug Burden Index), locally agreed thresholds should trigger stage escalation, pharmacist–GP co-review, and defined review frequency. Figure 1 visualises the diagnostic domains and stage assignment; Table 1 lists example measures and triggers.

Why staging matters: diseases are managed to match the intensity of the intervention with risk. A comparable strategy is feasible with the medication burden. For example, high anticholinergic exposure patients—older people with frailty or dementia—have a disproportionate risk profile that is lost when counting only the number of drugs taken. International data highlight the widespread use of high-potency anticholinergic drugs in groups with cognitive impairment and frailty, identifying a key exposure suitable for severity staging (e.g., none/low, moderate, high anticholinergic burden) [14]. Severity could also be staged with a composite including drug burden, interaction flags, red-flag combinations, and recent medication-linked events, with explicit thresholds to trigger escalation—low-intensity review for lower tiers and pharmacist-led, multivisit deprescribing plans for higher tiers [14]. For non-oral and long-acting agents, staging should also consider monitoring load and administration logistics (for example infusion visits and cold-chain requirements) so that apparent “de-pill” regimens are not misclassified as low burden.

**Figure 2:**
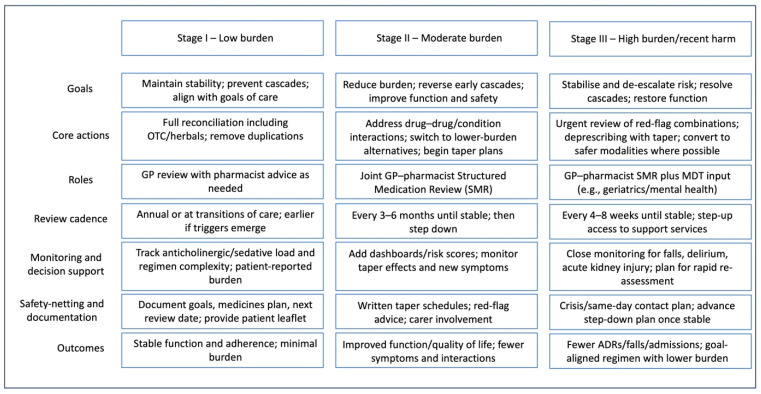
Stage-matched management pathway. Visual translation of stages into practical care: goals, core actions, roles, review cadence, monitoring/decision support, safety-netting/documentation, and outcomes for low, moderate, and high/recent-harm presentations. Emphasises GP–pharmacist Structured Medication Review (SMR), escalation to multidisciplinary team (MDT) for high burden, and treat-to-target plans prioritising function, safety, and reduced regimen burden. Examples should be tailored to local formularies, services and thresholds. Abbreviations: SMR, Structured Medication Review; MDT, multidisciplinary team; ADR, adverse drug reaction.

A relapsing–remitting course: like most long-term conditions, the medication burden fluctuates. A new diagnosis or step-up therapy produce “exacerbations” in regime complexity. Framing polypharmacy as a chronic condition transforms its management from sporadic to proactive by incorporating scheduled follow-ups after hospital discharge, time-limited trials of new drugs with gradual escalations, and clear stop dates. Notably, real-world studies confirm that deprescribing in primary care is feasible without compromising health outcomes, endorsing proactive review as a safe standard practice [15]. Systematic reviews further demonstrate that sustained improvements stem from comprehensive, longitudinal programs rather than isolated, opportunistic interventions [8]. Stage-matched management is summarised in in Table 2 and Figure 2.

## 4. Stage-Matched Management

Treat to target does not mean “less at all costs”. A condition lens rejects “the fewer the better” as an undifferentiated objective. Instead, it accepts function/safety related targets: reduce anticholinergic/sedative burden below a threshold value; eliminate high risk duplication; avoidable interaction resolution; and synchronize each medication with current goals of care. Current appropriateness guidelines (e.g., STOPP/START, Beers) aid the identification of discontinuation/initiation opportunities, but are underused [16]. Incorporating an evidence-based, patient-centred process of deprescribing—evaluation, shared prioritisation, cautious tapering, monitoring for withdrawal/recurrence—translates targets into safely sequenced steps rather than blunt discontinuations [17]. That is, problematic polypharmacy’s “diagnosis” invokes a structured pathway analogous to hypertension management: confirm, stage, intervene, and review to target [16,17].

Team-based care as standard of care involves framing polypharmacy as normalising pharmacist collaboration and Multi-Disciplinary Team (MDT) reviews, transforming collaboration from “referral if stuck” to “regular co-management”. Primary care clinicians typically report that deprescribing is simpler and more agreeable if pharmacists are integrated, particularly to discern interactions and to design taper plans; pharmacists then point to shared goals and shared understandings about roles [18,19]. Charting real world deprescribing interventions to the Behaviour Change Wheel clarifies multi-level levers—capability, opportunity, motivation—required for sustainable practice change, consistent with a chronic care model rather than an individual clinician’s enterprise [20]. Patients’ and GPs’ decisions depend on perceived necessity, worry about destabilising conditions, and workload/time restrictions—all susceptible to planned, staged review and shared discussion [21]. Hence, if unit of care is the regimen, not the prescription, team-based working is no longer a luxury [18,19,20,21].

The goals and preferences of patients focus the plan. Patients’ desire to reduce medications varies widely; some older adults prioritise symptom stability over reducing prescriptions, while others embrace simplification to enhance cognition, functional status, or daily activities. Realist evaluations of deprescribing show that patients are more likely to proceed when they understand the link between their symptoms and medications, know what to expect during tapering, and have clear measures of success [22]. An operational framework for polypharmacy as a chronic condition puts this into practice by setting specific goals (e.g., reducing daily falls), assessing patient preferences and risk tolerance, and establishing monitoring plans (e.g., weekly check-ins during benzodiazepine tapering). Patient preferences are shaped by effective clinician communication and high-quality shared decision-making, and framing polypharmacy as a chronic condition validates these discussions while challenging the assumption that long-term medications must continue indefinitely [23,24,25].

System design and continuity: sustained primary care relationships lead to more personalised prescribing, likely due to clinicians’ deep understanding of a patient’s pharmacological history and values [11]. Framing polypharmacy as a chronic condition elevates continuity as a core therapeutic component, achieved by designating clinicians responsible for medication stewardship, scheduling regular Structured Medication Reviews (SMRs) at set intervals, and implementing recall prompts following key care transitions. Changing prescriber behaviour requires addressing not only knowledge but also contextual factors like time, workflow, and incentives; recent studies identify drivers of deprescribing behaviour across professional groups, informing the design of supportive systems and feedback mechanisms [26,27]. To overcome limited time and competing priorities, practices should schedule pharmacist–GP joint SMRs with templated notes, protect short “add-on” review slots within routine clinics, and convene brief MDT huddles for high-risk patients.

Digital tools as enablers, not substitutes: a chronic care model embraces AI-enabled dashboards that distil and track the medicine burden—anticholinergic/sedative burden, interaction warnings, recent changes, taper schedules—alongside patient-reported outcomes (sleep, balance, thinking) and near-term risk predictions (for example falls or delirium). Medication review tools, if integrated into the EHRs (electronic health records) and used longitudinally, can support case finding (who to review now?), staging (what is the burden’s severity?) and targeting (what exposures contribute most to risk?) [13]. Machine-learning models trained on longitudinal EHR data—and, where available, pharmacogenomic profiles—can prioritise reviews, predict complications, and suggest stage upgrades, but should be locally validated and used transparently as decision support rather than automated decision-making. Prediction models for medication-linked events should undergo external validation and impact analysis embedded in routine EHR workflows to confirm clinical utility and transportability. High quality syntheses reaffirm that interventions with clinician education, pharmacist review, and clinical decision support optimise prescribing better than any single component in chronic care reasoning [28]. Technology should nudge and prioritise; clinicians should interpret, individualise, and share targets with their patients [13,27,28]. Digital therapeutics and personalised medicine (for example pharmacogenomic-guided dosing) can substitute or streamline drug regimens and thereby lower tablet counts; within our framework they are still counted within regimen burden and monitored with the same staging, targets, and follow-up.

Equity and person-centred outcomes: burden and harm from complex regimes differentially impact according to social determinants such as isolation, deprivation, and health literacy. Inclusion of polypharmacy in long-term condition registers permits proactive review with priority for individuals least likely to review themselves, aligned with population health goals and maximum benefit where most necessary. Improvement here is measured as much by function, safety, and quality of life that matters to people—few falls, better thinking clarity, more even gait—making medication stewardship concrete and relevant [12].

## 5. Discussion

The argument for reframing polypharmacy as a chronic condition rests on clinical logic (a persistent, worsening pattern that predicts harm), measurement (validated tools to stage risk), and practicality (systems and processes for safe, patient-centred outcomes). Clinically, a “chronic polypharmacy” diagnosis prompts a structured approach: confirming whether the current regimen likely causes symptoms or risks, staging severity using burden indices and red flags, setting shared goals with the patient (e.g., reducing falls or improving clarity), prioritising actions based on risk–benefit and patient preferences, and planning follow-up to prevent relapse. Conceptually, it shifts the focus from sporadic medication adjustments to ongoing stewardship, embodying the hallmarks of chronic disease management and paving the way for safer, more effective care (Table 1).

Prior perspectives have typically framed the problem as “problematic polypharmacy” to be identified and reduced, focusing on definitions and case-finding in multimorbidity rather than a condition-level pathway [2]. Deprescribing papers have clarified language and process—what deprescribing is and the steps to do it—but usually treat it as a discrete intervention rather than a longitudinal programme with diagnosis, staging and treat-to-target follow-up [7,8,17]. Evidence syntheses emphasise multi-component packages (education, pharmacist review, decision support) yet stop short of specifying when to escalate care or how to set regimen-level targets [28]. Our contribution is to integrate these strands into a chronic-condition model: a working definition, diagnostic domains, explicit staging cues and action thresholds, with continuity/equity as design principles and cascades/anticholinergic load as exemplar phenotypes to operationalise risk [5,11,12,14,16].

### 5.1. Implications for Clinical Practice

A recognition of polypharmacy as a chronic disease redirects clinical activity from rapid simplification of lists of medications to long-term stewardship with a focus on diagnosis, agreed goal staging, and planned follow-up. In practice, it means applying to pharmacy optimisation the habits and tools of chronic disease management—continuity, recall systems, multidisciplinary input, and patient reported outcome. It is also foreshadowed in the work on deprescribing: brief process steps, taking account of patient intent, an emphasis on recurrent review rather than a one-off activity [7,8,28].

The first implication is a diagnostic one. Clinicians should regularly consider medication burden as a cause when non-specific symptoms—dizziness, falls, confusion, gastrointestinal symptoms—present in older or multimorbid individuals or if care usage suddenly escalates, and these findings should trigger immediate review irrespective of medication count [1,3,4]. Prescribing cascades serve as a critical warning sign, indicating an unchecked regimen, and warrant the same urgency as any flare-up in a chronic disease trajectory [5]. Non-prescribed agents commonly contribute to cascades (for example sedating antihistamines, NSAIDs); clinicians should ask specifically about these and document them within structured medication reviews. Anticholinergic and sedative burden, for example, should be regularly reviewed in older adults and cognitively impaired or frail individuals, due to the persistent link with falls and functional decline in a number of countries among real world cohorts [14].

The second is to replace a blunt “less is more” dogma with treat-to-target medication stewardship. Targets should be functional or safety oriented—lowering anticholinergic burden to less than a threshold value, eliminating patently incorrect combinations, resolving duplicated indications, or attacking symptom goals relevant to the individual—rather than just a reduction in number of medications. At times polypharmacy is necessary—for example short-term intensification during acute illness—so decisions should aim for net clinical benefit with explicit “step-down” plans and review dates once stability returns. Deprescribing protocols based on evidence offer a safe pathway to change: assess indication and risk, agree with the individual about priorities, taper slowly where necessary, monitor withdrawal or symptom rebound, and refine iteratively [17]. Such programs can reduce exposure with no worsening of health-related outcomes, deserving more active default deprescribing among eligible candidates [15]. Medication review should therefore be ordered and conducted as a standard “clinic” for this condition, with forms including burden indices, goals of care, agreed adjustments, and plans for safety-netting, and may be delivered via community-pharmacy or home-based reviews when access barriers are present [27].

Third, behaviour change science shows that practice is maintained if capability (education regarding tapering and risk talk), opportunity (time, workflow access to pharmacists), and motivation (outcome feedback, shared goals) are introduced [20]. Even where clinicians perceive that a medication is inappropriate, qualitative work warns that organisational barriers—time pressure, divided attention with unclearly organised or missing records, ambiguous responsibility—can thwart intentions; defining polypharmacy as a chronic condition sanctions avoided time and shares responsibility to eliminate these barriers [25,26].

Fourth, continuity and equity should be core design principles. Continuity of relationship in primary care leads to more appropriate prescribing, almost certainly because clinicians who have a good grasp of the individual and their pharmacological trial history can better strike a balance between trade-offs and recognise cascades early [11]. In the population, patients living with deprivation, isolation, or low health literacy have a higher risk of medication related harm but a lower initiation of review likelihood; including polypharmacy in long-term condition registers enables proactive outreach and tailoring to maximise benefit with a minimum of unintended inequities [12].

Lastly, surveillance and safety netting should be similar to other chronic conditions. Improvement should be tracked with some pragmatic markers—burden scores (e.g., anticholinergic burden), presence of potentially inappropriate medications, frequency of unplanned changes to regimen, and patient-reported outcome measures regarding function and quality of life. Because of the well-established polypharmacy–adverse event correlations among older adults, falls and emergency department utilisation are sensible sentinel end-points to track over time [1,3,4]. When tapering is being undertaken, clinicians should be definite about what success is (e.g., better thinking or fewer falls at night within four weeks), how often to review, and what to do if symptoms don’t resolve. Above all, deprescribing should never be framed as a challenge to compliantly take medication but as a collaboratively owned treatment trial with early reintroduction or alternate approaches if agreed objectives are not achieved [22,23,24].

In summary, clinical practice for managing polypharmacy as a chronic condition has practical, real-world conclusions: suspect early where presentations are non-specific; stage risk with structured tools; respond with treat-to-target optimisation through commissioned, template led reviews; make collaboration with pharmacists a routine; base decisions on preference in the patient; protect the work with continuity recall; and screen for things that matter in their outcomes. Done regularly, this converts medicine optimisation from a periodic tidy to the consistent preventative person-centred care that long term conditions demand [7,8,11,12,15,16,17,18,19,20,21,27,28].

### 5.2. Implications for Education and Training

Redefining polypharmacy as a chronic disease also requires a simultaneous redesign of clinician education. Teaching often remains focused on treating single diseases and drug pharmacology despite projected harm that accrues with complex regimens accumulating among older and multimorbid individuals [1,2,3,4]. Medication burden is to be perceived as a stageable, diagnosable problem with avoidable complications only if diagnostic thinking, communication, and teamworking regarding medications is learned and assessed as a core clinical competence, not an elective afterthought. That begins with placing polypharmacy explicitly in the differential diagnosis for frequent non-specific presentations—dizziness, falls, fatigue, confusion—and with teaching students to look for temporal correlations between symptoms and recent changes in therapy, including prescribing cascades that disguise themselves as disease progression [3,4,5]. Case-based learning and simulation should present such patterns to students, asking them to generate medicine-first hypotheses before anchoring onto organ specific explanations.

Competence should progress from recognition to organised management. Students and trainees should be familiar with appropriateness frameworks (STOPP/START, AGS Beers), and with composite burden scales that take a list and make it into risk, such as anticholinergic or sedative loading indices [13,16]. These should be standardised in skills teaching and in exams (ward-based reviews, OSCE stations), instead of being reserved for specialist modules. Information literacy is also necessary: learners should be practised with EHRs and with clinical decision support to discern interactions, to highlight duplicate prescribing, and to plot taper plans within templatised “structured medication reviews,” reflecting how safe optimisation is implemented in community care [27,28]. Education should stress that decision support supplements, but does not replace, clinical judgement or shared decision making.

Interprofessional education is central. Collaborative deprescribing is effective and acceptable, and should be translated into pharmacists codesigning and codelivering education, with simultaneous simulation of medication reviews and team assessments that require role clarification and shared planning [18,19]. Aligning teaching activity with behaviour change frameworks can ensure that they work to influence capability (how to taper and monitor), opportunity (workflow, referral routes to pharmacists), and motivation (feedback and goal alignment), to match the multi component nature of successful implementation in practice [20,28]. Clinical supervisors should explicitly operationalise the consultation micro skills that enable deprescribing—eliciting a person’s goals, negotiating risk trade-offs, planning safety netting—to close the gap between classroom rhetoric and clinical reality.

Education should also recognise that preference and belief routinely determine whether deprescribing is effective. Older adults’ intentions to reduce medication switches hinge on perceived necessity and harm, identity attachment to medication, and trust in the tactic; accordingly, students should be trained to enquire about and document such variables and to offer time limited experiments with clear success and reintroduction criteria [21,22,23,24]. It is a courtesy of communication but a clinical outcome determinant, and should be apparent in marking schemes and work-based exams.

Finally, educational programs should embed equity and quality improvement perspectives. Training on social determinants should move beyond theory by tasking trainees with tailoring medication reviews for individuals facing deprivation, isolation, or low health literacy, evaluating whether improvements enhance function and quality of life alongside pharmacometric measures [12]. Students should engage in supervised micro-quality improvement (QI) cycles, applying evidence-based deprescribing protocols and monitoring outcomes, building on evidence that optimisation is feasible without compromising health if plans are clear and follow-up is consistent [15,17]. Faculty development is crucial, as supervisors’ culture and local practices shape clinical habits; training educators to use shared templates, seek pharmacist input, and prioritise review time aligns teaching with evidence-based standards [26,27,28]. Collectively, these educational reforms—emphasizing diagnostic awareness, structured tools, interprofessional collaboration, preference-sensitive communication, continuity, equity, and QI—equip future clinicians to manage polypharmacy proactively, longitudinally, and with a person-centred focus.

### 5.3. Policy and System Implications

Framing polypharmacy as a chronic condition has implications for organising care, securing funding, and defining success. Policies should prioritise the registration and recall of high-risk patients, as with other long-term conditions, using electronic registries with practical criteria (e.g., ≥5 medications alongside frailty or recent hospitalization) and automated composite measures like anticholinergic or sedative burden [13,14]. Recall should be prompted by care transitions or the addition of drugs or doses that increase pharmacological burden, recognizing that most regimen “exacerbations” occur post-hospital discharge or due to guideline layering [5,14,15]. Continuity of care should be safeguarded, as sustained primary care relationships are linked to more tailored prescribing and safer deprescribing; thus, commissioning and workforce strategies should incentivize designated clinicians for medication stewardship within practices or primary care networks [11].

Secondly, systems should initiate SMRs as a standard, template-based approach. Evidence syntheses verify that multi component interventions—educating the clinician, pharmacist consultation, decision support, and audit/feedback—outperform single interventions; payers should therefore reimburse multi component packs rather than single components and evaluate them by regimen level rather than simplistic medication number [8,28].

Third, integration of pharmacists should move beyond being optional to being a norm. Policy should include funding for pharmacist sessions in primary care to jointly execute SMRs, jointly design taper schedules, and follow up with a specified division of labour among general practitioners, community pharmacists, and specialists [18,19]. As behaviour to deprescribe is determined by capability, opportunity, and motivation, support for implementation should include learning to taper and communicate risk effectively, organisational adjustments to facilitate easily available pharmacist advice, and high-frequency feedback on results—both a direct translation to levers for behaviour demonstrated to be necessary for primary care [20,26]. Mechanisms for contracting can support such an arrangement by connecting remuneration to dual review, signed decisions agreed jointly, and date-specific review.

Fourth, systems should include equity in medicines optimisation, embedding practical steps such as translation and interpreter services, caregiver involvement, longer appointments for high social risk, proactive recall of those unlikely to self-present, and group education clinics co-led by pharmacists. Isolation, deprivation, and low health literacy—important social determinants—influence exposure to complex regimens as much as ability to take part in reviews; registries should therefore include social risk fields and trigger outreach, translation, involvement of caregivers, and longer consultations where needed [12]. Community-pharmacy summaries and brown-bag checks can be used opportunistically at vaccination, blood-test, and long-term-condition visits to capture those who struggle to book separate reviews. Success measures should include patient reported treatment burden and functional outcomes alongside pharmacometrics measures so that reduction of risk is never a trade-off against what is valuable to the individual [12,17].

Fifth, digital infrastructure should support—but never supersede—clinical judgment. Lists of interactions generated passively are inadequate; systems should also signal risk focused summaries (e.g., anticholinergic/sedative burden, red flag combinations, recent unplanned changes) and enable easy documentation of agreed decisions, taper steps, and follow-up intervals [13,27]. Practice or network level dashboards can track a few population indicators—proportion above an agreed burden threshold, prevalence of potentially inappropriate medications, time since last SMR, completion rates of deprescribing without symptom rebound—to support quality improvement and commissioning [13,16,28].

Finally, education and governance should be matched with policy intent. Undergraduate and postgraduate teaching should cover polypharmacy as a diagnostic and management competence, and local governance should audit cascades, high risk combinations, and review periodicity with learning responses rather than blame if issues are identified [6,7,8,16]. Taken as a package, these policy moves—registration and recall, commissioned SMRs, integration of pharmacists, equity first design with assistance from assistive digital tools, matched educating—change medicine optimisation from occasional tidying to a logical, measurable long term condition programme consistent with the literature on what produces safer, more appropriate prescribing over time [8,11,13,14,15,16,18,19,20,26,27,28].

### 5.4. Future Directions

To treat polypharmacy as a chronic condition, we must translate this concept into actionable diagnostic criteria. Research should focus on developing and testing a practical diagnostic framework beyond pill counts, using multicentre longitudinal cohorts and registry-based studies to derive and validate diagnostic criteria and staging thresholds [13,14,15,16,17]. Prospective studies should evaluate staging and phenotype models—such as sedation-linked falls, anticholinergic-related cognitive decline, or cardiorenal risks from NSAIDs—assessing how stage-specific interventions impact outcomes like falls, delirium, emergency visits, functional status, and quality of life in high-risk older and multimorbid populations [1,3,4,14]. To establish effectiveness, pragmatic cluster randomised or stepped-wedge trials should compare stage-matched, GP-pharmacist co-managed care versus usual care, with primary outcomes including falls, delirium, emergency use, function, quality of life, and cost-effectiveness. Because effective optimisation requires a multifaceted approach, pragmatic trials should compare standard care with packages integrating pharmacist-led structured medication reviews, decision support, continuity of care, and audit/feedback with pre-specified patient-centred endpoints and health-economic evaluation, while monitoring for symptom recurrence post-deprescribing [8,15,27,28].

Implementation science can help identify drivers of success. Electronic health records should evolve beyond static interaction lists to include risk-focused summaries and templates that streamline the documentation and tracking of care elements, such as agreed goals, tapering plans, and safety-netting measures [13,27]. Commissioning should prioritize longitudinal programs over one-off initiatives, recognizing that behaviour change hinges on enhancing clinicians’ and patients’ capability, opportunity, and motivation, with pharmacist integration as an essential component, not an optional extra [18,19,20,21,28]. Equity must be embedded by design, incorporating registries, recall systems, document translation, caregiver involvement, and extended consultations for those with higher social risks, alongside standardized reporting of patient-reported burden and functional outcomes in addition to pharmacometric measures [11,12,17].

## 6. Conclusions

We must embed medication-related considerations into diagnostic reasoning for non-specific symptoms, equipping clinical trainees to apply appropriateness criteria and burden indices at the point of care, supported by interprofessional training and supervised micro-quality improvement (QI) cycles that achieve progress without adverse effects. Faculty development should synchronise the hidden curriculum—time allocation, standardised templates, and collaborative teamwork—with evidence-based, practical approaches. Ultimately, redefining polypharmacy as a chronic condition shifts medication management from occasional list updates to a structured program encompassing diagnosis, staging, goal-setting, and follow-up. This integrates clinical judgment with real-world data, emphasizes continuity and pharmacist collaboration, prioritizes patient preferences, and provides a measurable policy framework for safer, more effective care. The key challenge lies in implementation: establishing registries, mandating structured reviews, embedding supportive digital tools in routine practice, and ensuring systems deliver outcomes that benefit patients and are sustainable for clinicians.

## Figures and Tables

**Table 1:** Diagnosing and staging “polypharmacy as a chronic condition”.

Domain	What to Look for at the Bedside	How to Measure (Examples)	Trigger/Threshold (Locally Agreed)	What to Do Next
Persistence & progression	Regimen sustained over months; step-wise growth after admissions/guideline layering	Medication reconciliation over prior 6–12 months	Sustained multi-drug exposure with recent escalation	Apply condition label; enrol in structured medication review (SMR)
Regimen burden	Sedative/anticholinergic load; interaction density; duplications	Anticholinergic/sedative burden indices; interaction checkers; STOPP/START, AGS Beers	Burden above local “high-risk” threshold or ≥1 red-flag combination	Prioritise high-risk exposures for taper/substitution in order of harm
Complications	Falls, delirium/confusion, orthostasis, AKI, GI bleed, functional/cognitive decline	Recent ED/ward contacts; functional tests; cognitive screens; labs	Any plausible medication-linked event in last 6–12 months	Upgrade staging; move to urgent stabilisation plan and MDT input
Prescribing cascades	New drug started to treat a side-effect of an existing drug	Timeline linking symptom→new prescription	Confirmed or strongly suspected cascade	Plan cascade reversal (de-escalate offending drug first)
Contextual risk	Frailty, renal/hepatic impairment, poly-clinician care, low health literacy, isolation	eFI/clinical frailty, eGFR/LFTs; social history	High clinical or social vulnerability	Adapt communication, involve carers, extend appointment time
Staging & cadence	Stage I: controlled burden	Low burden; no recent complications	Annual SMR	Maintain; prevention focus
	Stage II: unstable burden	Rising burden or early warning symptoms	SMR every 3–6 months	Targeted deprescribing; pharmacist co-management
	Stage III: decompensated	Recent serious event plausibly drug-related	Immediate review	Rapid stabilisation; MDT plan and safety-netting

Abbreviations: SMR, structured medication review; MDT, multidisciplinary team; AKI, acute kidney injury; eFI, electronic frailty index; eGFR, estimated Glomerular Filtration Rate; ED, emergency department.

**Table 2:** Stage-matched management: from stabilisation to maintenance.

Stage	Clinical Picture	Priority Risks	Core Actions (Treat-to-Target)	Who Does What	Review Interval	Outcomes to Track
I. Controlled burden	Stable list; low burden indices; no recent events	Drift/accumulation; silent interactions	Agree goals; check necessity/indication; remove low-value meds; document “if-this-then-that” triggers	GP: stewardship & shared decisions; Pharmacist: interaction/burden check; Patient/carer: goals & monitoring	12 months (or after major changes)	Burden index stable/↓; PIMs ↓; patient-reported treatment burden ↓
II. Unstable burden	Rising burden or new non-specific symptoms (fatigue, dizziness, confusion, falls)	Sedative/anticholinergic load; cascades	Sequence deprescribing (highest harm first); taper plans; alternatives; written plan & safety-net	GP & Pharmacist: joint SMR; Nurse/Allied: function & falls review	3–6 months	Fewer red-flag combinations; fewer unplanned changes; function/clarity ↑
III. Decompensated	Recent serious event (fall with injury, delirium, AKI, GI bleed) plausibly medication-linked	Re-injury, withdrawal phenomena, undertreated symptoms	Rapid stabilisation: stop/switch high-risk drugs, reverse cascades, close monitoring; involve carers	MDT huddle within 1–2 weeks; GP leads; Pharmacist co-leads taper; Carer engaged	1–4 weeks until stable, then step down	Event-free interval ↑; ED use ↓; symptom goals achieved without rebound

Abbreviations: GP, general practitioner; SMR, structured medication review; AKI, acute kidney injury; PIMs, potentially inappropriate medicines; ↑/↓, increase/decrease; ED, emergency department.
